# A Retrospective Clinical Analysis of Pain and Spasticity Outcomes Following Gravity-Support Exoskeleton Therapy in Chronic Stroke

**DOI:** 10.3390/jcm15062099

**Published:** 2026-03-10

**Authors:** Mirjam Bonanno, Desiree Latella, Paolo De Pasquale, Mauro Botindari, Antonino Lombardo Facciale, Angelo Quartarone, Rosaria De Luca, Giovanni Morone, Rocco Salvatore Calabrò

**Affiliations:** 1 IRCCS Centro Neurolesi Bonino-Pulejo, 98124 Messina, Italy; mirjam.bonanno@irccsme.it (M.B.); desiree.latella@irccsme.it (D.L.); mauro.botindari@irccsme.it (M.B.); antonino.lombardo@irccsme.it (A.L.F.); angelo.quartarone@irccsme.it (A.Q.); rosaria.deluca@irccsme.it (R.D.L.); roccos.calabro@irccsme.it (R.S.C.); 2 Santa Lucia Foundation, Scientific Institute for Research, Hospitalization and Health Care (IRCCS), 00179 Rome, Italy; giovanni.morone@univaq.it; 3 Department of Life, Health and Environmental Sciences, University of L’Aquila, 67100 L’Aquila, Italy

**Keywords:** upper limb, robotic-assisted therapy, post-stroke pain, shoulder pain, neurorehabilitation

## Abstract

**Background:** Post-stroke pain (PSP), particularly shoulder pain, is frequent and often underdiagnosed, limiting rehabilitation adherence and functional recovery. Current pharmacological and physical treatments offer only partial relief. Robotic-assisted therapy (RAT), such as the gravity-supporting Armeo^®^ Spring exoskeleton, delivers intensive, task-specific training with visual 2D feedback that may also alleviate PSP while enhancing motor outcomes. This study investigates whether RAT performed with the Armeo^®^ Spring reduces upper-limb PSP in chronic stroke patients versus conventional therapy and evaluates its effects on motor function and functional independence. **Methods:** In this retrospective parallel group study, 32 chronic post-stroke patients (8 females and 24 males with a mean age of 57 ± 11.74) were allocated to two groups: 16 received upper-limb RAT with the Armeo^®^ Spring, a gravity-supporting exoskeleton, (RAT group) and 16 underwent conventional rehabilitation (CR). The RAT group completed one-hour sessions 6 days/week for 8 weeks, performing 2D/3D gamified tasks targeting shoulder, elbow and forearm movements. The CR group received an equivalent amount of standard therapy, including passive/active-assisted mobilization, Bobath-based neuromuscular facilitation and reaching exercises. **Results:** Both the Armeo^®^ Spring and conventional therapy groups showed significant reductions in post-stroke pain (RAT *p* < 0.001 and conventional rehabilitation *p* = 0.004) and improvements in upper-limb motor function and functional independence (both *p* ≤ 0.002). Spasticity in the impaired limb decreased modestly in the RAT group (*p* = 0.031), with no significant between-group differences in pain or spasticity change (*p* = 0.437; *p* > 0.05, respectively). **Conclusions:** Gravity-support exoskeleton training reduced upper-limb spasticity, and no statistically significant between-group differences were observed compared with conventional physiotherapy for pain, mobility, and functional independence. Although clinical outcomes improved, health-related quality-of-life domains showed heterogeneous trajectories, underscoring the complexity of perceived health changes during chronic stroke rehabilitation. Larger randomized controlled trials incorporating neurophysiological and kinematic endpoints and longer follow-up are warranted to confirm effectiveness, particularly in chronic stroke and durability.

## 1. Introduction

Stroke is recognized as brain damage due to an acute focal injury of the central nervous system (CNS) by a vascular damage, including ischemic and hemorrhagic etiologies. It is considered one of the major causes of disability and death worldwide [1]. In 2017, it was estimated that approximately 1.12 million people in Europe were affected by cerebrovascular disorders due to ischemic or hemorrhagic events [2]. Regarding the motor consequences, post-stroke patients can manifest altered motor patterns in the contralesional upper and lower limbs, due to paralysis and/or spasticity [3]. In addition to motor and sensitive deficits, pain after stroke is another common condition, which is associated with worsened health outcomes [4,5,6,7].

Pain can be a significant barrier to adherence to the rehabilitation pathway, preventing stroke survivors from reaching their maximal functional potential. The prevalence of post-stroke pain (PSP) has been reported in 66% of cases, although this estimate can depend on study methods [8]. According to a prospective, cross-sectional, multicentre study, the authors [9] documented that the prevalence of PSP in the acute stage (within 14 days of stroke) is around 14%; in the subacute stage (15–90 days after stroke), it is 42%; and lastly, in the chronic period (>90 days after stroke), it was reported at 31%. Stroke survivors who experience pain have greater rates of depression, suicide rates, disability, fatigue, cognitive impairment, and functional decline compared with those without pain after stroke [4,6,8,10]. The most common localization of PSP is the shoulder, likely due to the muscular imbalance in the upper limb [11]. It not only exacerbates physical discomfort but also can represent a significant barrier to rehabilitation approaches, reducing adherence to the treatment and patients’ motivation [12]. In addition to pain, post-stroke patients tend to manifest spasticity, which is in turn one of the most common risk factors for the development of PSP. According to Wissel et al. [13], spasticity is often associated with pain in stroke patients. The authors reported that 72% of the patients with spasticity experienced pain, while only 1.5% of non-spastic patients exhibited pain syndrome. Spasticity is associated with 60% of cases of shoulder pain, 100% of cases of elbow pain, and 33% of cases of wrist pain. The potential relationship between pain and spasticity may be explained by the fact that stretching a spastic muscle can cause micro-disruptions of muscle fibers and trigger the release of algesic substances, which in turn activate muscle nociceptors and generate nociceptive pain [8].

Despite the high occurrence of PSP in addition to spasticity, it is often underrecognized and, consequently, undertreated [8,9]. A wide range of therapeutic strategies has been explored to manage PSP, including oral medications, orthotic devices, botulinum toxin injections, and various forms of physical therapy. Although each of these interventions has shown partial effectiveness, none has yet proven to be a definitive or comprehensive solution [14].

A promising avenue in stroke rehabilitation is the field of robotic-assisted therapies (RAT). RAT offers intensive, repetitive, task-specific training that can stimulate neuroplasticity, even in chronic stages [15]. Robotic rehabilitation can provide a guide for motor recovery, providing controlled, repetitive, and variable patterns. In this way, the increased therapy dosage, intensity, number of repetitions, and execution of exercises can promote plasticity and functional recovery. RAT can be considered a useful rehabilitation tool to generate controlled multisensory stimulation of the patient. Different from gait robotic systems, upper limb robotic devices are often equipped with a 2D non-immersive virtual reality monitor or serious game. In this sense, these devices promote multisensory stimulation through augmented audio–visual feedback that can cause patient motivation and enjoyment [16] and might modulate attentional resources that might be crucial for motor learning [17], functional recovery [18] and pain perception [19]. Despite the proven effectiveness of robotic devices for upper limb motor recovery in post-stroke patients, the available data on the efficacy of robotic-assisted rehabilitation as a treatment option aimed at reducing pain in stroke patients with PSP are limited, as reported by Gnasso et al. [20].

The primary aim of this study is to evaluate the effects of robotic-assisted therapy for PSP and spasticity in upper limbs for patients with chronic stroke, using a gravity-supporting exoskeleton system (Armeo^®^ Spring), compared to a control group, who performed conventional rehabilitation therapy. A secondary objective of the study is to investigate the functional effects of RAT, in terms of movement and quality of life, compared to conventional rehabilitation therapy.

## 2. Materials and Methods

### 2.1. Study Design and Population

This retrospective parallel group study included post-stroke patients admitted to the Intensive Neurorehabilitation Unit of IRCCS Centro Neurolesi “Bonino-Pulejo” (Messina, Italy) between June 2023 and May 2024. Clinical and rehabilitation data were extracted from electronic medical records and, when applicable, from the Armeo^®^ Spring device logs. Eligible participants were adults (>18 years) with a confirmed diagnosis of ischemic or hemorrhagic stroke in the chronic phase (≥6 months post-event), clinically stable, and with sufficient cognitive ability to understand and follow instructions. Exclusion criteria comprised other major neurological disorders (e.g., Parkinson’s disease, multiple sclerosis, epilepsy), severe cognitive or communication impairments, and unstable medical conditions (e.g., decompensated cardiac or respiratory disease, active cancer). Patients were retrospectively allocated to one of two cohorts, according to the rehabilitation program received as part of standard clinical care: RAT using the Armeo^®^ Spring exoskeleton (*n* = 16), or conventional rehabilitation (CR) based on therapist-guided upper-limb exercises (*n* = 16). As this was an exploratory retrospective study, the sample size was based on all eligible patients within the study period, and no priori power calculation was performed. The study complied with the ethical standards of the 1964 Declaration of Helsinki and received approval from the local Ethics Committee (IRCCS-ME-CE 18/2024).

### 2.2. Procedures

The included post-stroke patients were divided into two groups with comparable demographic characteristics (age and sex). Patients received either RAT using the Armeo^®^ Spring exoskeleton or CR. All patients underwent an upper-limb rehabilitation program consisting of one-hour sessions, administered six times per 8 weeks. In all participants, the intervention was delivered on the clinically affected side (impaired upper limb).

Each session was conducted individually under the supervision of a trained physiotherapist, following standardized clinical protocols routinely adopted at the Neurorehabilitation Unit. Following completion of the rehabilitation interventions, we retrospectively screened clinical records to identify patients meeting the predefined inclusion/exclusion criteria. Eligible patients were then selected and matched between groups, according to the study criteria.

Motor and cognitive outcomes were retrospectively collected at admission and discharge from clinical records. All evaluations had been performed as part of standard care by a multidisciplinary team including a neurologist, a physiatrist, a physiotherapist, and a psychologist. Clinical evaluations were performed as part of routine standard care using standardized and validated scales by a multidisciplinary team. Due to the retrospective design, formal assessor blinding to treatment allocation was not implemented. Primary outcomes included changes in pain and spasticity measures, assessed through standardized clinical scales (e.g., the Numerical Rating Scale, Modified Ashworth Scale, Motricity Index). Secondary outcomes included changes in mobility, quality of life, and functional independence (e.g., Motricity index, Short Form-36, Functional Independence Measure). The baseline comparability between groups was verified for demographic and clinical variables (age, sex, stroke type, lesion side, and baseline scores). Records with incomplete pre- or post-treatment data were excluded from the analysis. All data were anonymized and organized in a dedicated database for subsequent statistical analysis.

### 2.3. Outcome Measures

Before (T0) and after (T1) the rehabilitation program, all patients underwent a comprehensive clinical evaluation conducted by a multidisciplinary team as part of routine practice. The assessment protocol included both primary and secondary outcome measures, capturing pain, spasticity, motor function, functional independence, and quality of life.

Pain intensity was evaluated using the Numeric Pain Rating Scale (NPRS), a widely adopted 11-point scale where patients rate their perceived pain from 0 (“no pain”) to 10 (“worst imaginable pain”) [21]. Muscle tone and spasticity were assessed through the Modified Ashworth Scale (MAS) [22], which classifies resistance to passive movement on a 5-point ordinal scale, ranging from 0 (no increase in tone) to 4 (rigid limb). Together, these measures provided a clinical picture of the pain and hypertonia commonly observed in post-stroke conditions.

Upper-limb motor function was examined using the Motricity Index (MI) [23], focusing on three representative movements, pinch grip, elbow flexion, and shoulder abduction, to quantify residual strength on a 0–100 scale, with higher scores indicating greater motor recovery. Overall functional independence was determined by the Functional Independence Measure (FIM) [24], which evaluates performance in activities of daily living through 18 items scored from 1 (total dependence) to 7 (complete independence), thereby encompassing both motor and cognitive dimensions of autonomy.

Finally, the patients’ perceived quality of life was explored using the Short Form-36 Health Survey (SF-36) [25], a multidimensional tool assessing eight domains of physical and mental health. This instrument offered a broader perspective on the psychosocial impact of rehabilitation and the patients’ overall well-being.

Changes observed between T0 and T1 across these measures were used to quantify rehabilitation outcomes and to compare the effects of robotic-assisted therapy with the Armeo^®^ Spring and conventional rehabilitation within the chronic post-stroke population.

### 2.4. Robotic-Assisted Training

Patients in the RAT group underwent robotic-assisted therapy using the Armeo^®^ Spring (Hocoma AG, Zurich, Switzerland), a gravity-supporting exoskeleton specifically designed for upper-limb rehabilitation. The Armeo^®^ Spring is integrated with a two-dimensional virtual reality (VR) environment that provides real-time visual feedback and gamified exercises. This setup enhances patient engagement and allows clinicians to monitor quantitative parameters such as range of motion, force, and coordination. Exercise difficulty can be dynamically adjusted through modifications in weight arm support, and increasing the level of difficulty of each exercise, making the training session more challenging.

Each session began with device calibration and adaptation to the patient’s anthropometric characteristics. The physiotherapist defined the active workspace and selected the most appropriate exercises, based on motor abilities, progressively modifying task parameters as recovery advanced. The typical training schedule consisted of one-hour sessions, six days per 8 weeks, with all sessions supervised by a physiotherapist experienced in robotic-assisted rehabilitation.

The robotic training sessions included a set of gamified 2D and 3D exercises routinely used in clinical practice to promote upper-limb mobility and coordination. Among the most frequently performed activities were Balloon, Roll the Ball, Clean the Ocean, Fly High, and Goalkeeper, each targeting different joint movements such as shoulder flexion–extension, abduction–adduction, elbow flexion–extension, and forearm pronation–supination, respectively. These tasks provided visual and auditory feedback to enhance patient engagement and support the motor learning process.

### 2.5. Conventional Rehabilitation Training

Patients who received CR were given the same global treatment time as the RAT group. CR consisted of therapist-guided sessions, including passive and active-assisted mobilization of the upper limb, neuromuscular facilitation, and proprioceptive and postural control exercises based on the Bobath concept. Functional reaching and grasping activities were also incorporated to promote upper-limb coordination and task-oriented recovery. All sessions were conducted individually by experienced physiotherapists, following the standard clinical practice of the Neurorehabilitation Unit.

### 2.6. Statistical Analysis

In both groups, RAT and the control, the baseline (pre-treatment) demographic and clinical characteristics were analyzed, including group, sex, age, education, etiology (ischemic vs. hemorrhagic), years since onset, Montreal Cognitive Assessment (MoCA), and affected side. Demographic variables were summarized for all participants (All), for the RAT group, and for the CR group. Continuous variables (age, education, years since onset, MoCA) were reported as mean ± standard deviation (SD) when normally distributed and compared between groups using independent-samples *t*-tests; when equality of variances was violated (Levene’s test, *p* < 0.05), the Welch correction was applied. When data were not normally distributed, Mann–Whitney U tests were used. Categorical variables (sex, etiology, affected side) were summarized as counts and percentages and compared between groups, using the chi-squared test (χ^2^) or Fisher’s exact test when expected cell counts were <5.

Clinical outcome measures collected pre- and post-treatment (after n sessions) included the NPRS, FIM, the eight domains of the SF-36 (Physical Functioning, Role Physical, Bodily Pain, General Health, Vitality, Social Functioning, Role Emotional, and Mental Health), and the two summary scores of the SF-36: the Physical Component Summary (PCS) and the Mental Component Summary (MCS). Additional outcomes included the MI and the MAS for the impaired and unimpaired limbs.

Given the small sample size and the ordinal or non-Gaussian nature of several variables, all longitudinal analyses were performed using non-parametric tests. For each scale and for the All, RAT, and CR groups, medians and interquartile ranges (Q1–Q3, 25th–75th percentile) were reported at T0 and T1, together with the change score Δ = T1–T0 (median [Q1–Q3]). Within-group pre–post differences (T0 vs. T1) were tested with the Wilcoxon signed-rank test. Between-group comparisons at T0, at T1, and on Δ (RAT vs. CR) were assessed using the Mann–Whitney U test. Exact *p*-values are reported, and those <0.05 are highlighted in bold in the tables.

Given the clinical and exploratory nature of the study and the number of analyzed scales, primary analyses were not adjusted for multiple comparisons and should be interpreted cautiously. All analyses were two-tailed and performed in MATLAB R2022a (The MathWorks, Natick, MA, USA). Moreover, given the limited sample size, the study had sufficient power to detect large between-group effects (Cohen’s d ≈ 1.0).

## 3. Results

**Figure 1:**
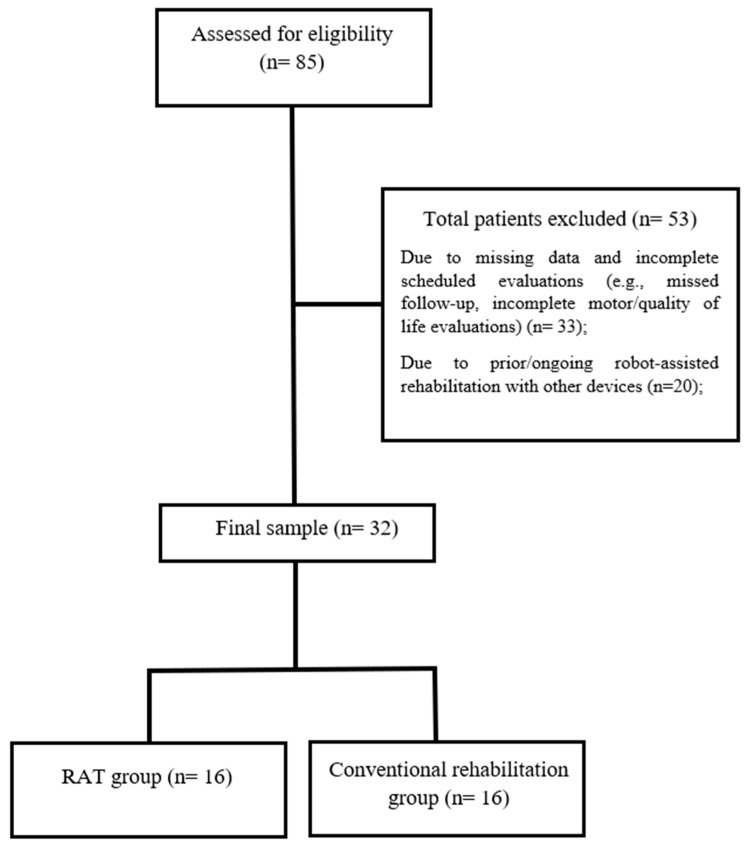
Flowchart of patient selection process. For detailed demographic and clinical characteristics of the sample, please refer to Table 1.

The medical records of 85 patients suffering from chronic stroke were included in the analysis utilizing electronic recovery system data. The final sample consisted of 32 patients, allocated in the RAT group (*n* = 16) or in the CR group (*n* = 16), who completed the rehabilitation process without reporting any side effects. The patient selection process is summarized in Figure 1.

At T0, there were no significant differences between the RAT and CR groups in any of the recorded variables (*p* > 0.05 for all) (see Table 1).

### 3.1. Primary Outcome Results

**Figure 2:**
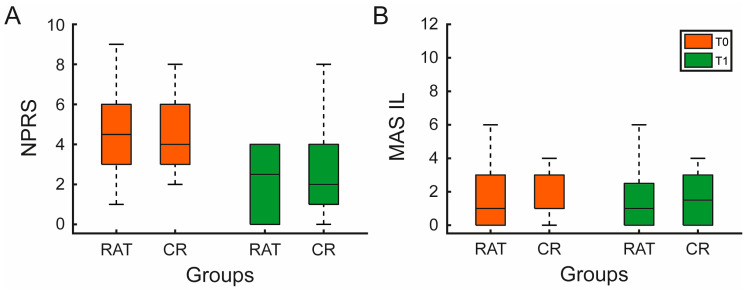
Pain and spasticity outcomes in experimental and control groups. Boxplots illustrate Numeric Pain Rating Scale (NPRS) scores (**A**) and Modified Ashworth Scale for impaired limb (MAS IL) scores (**B**), for the experimental (RAT) and control (CR) groups at baseline (T0, orange) and post-intervention (T1, green). For both panels, boxes represent the interquartile range (Q1–Q3), the horizontal line marks the median, and whiskers extend to the most extreme observed values within 1.5 × interquartile range.

A significant reduction in NPRS was observed in both groups after treatment (RAT: median Δ = −2.0 [−3.5–−1.5], *p* < 0.001; CR: Δ = −2.0 [−3.0–−1.0], *p* = 0.004, see Figure 2A and Table 2). No between-group differences were found at T0, T1, or in Δ (*p* = 1.000, 0.603, and 0.437 respectively; see Table 2).

Spasticity, measured by the MAS, showed a small but significant reduction in the impaired limb (RAT: *p* = 0.031), while changes in the unimpaired limb were negligible (*p* = 1.000), see Figure 2B and Table 2. No significant differences between groups were found for Ashworth scores (*p* > 0.05).

### 3.2. Secondary Outcome Results

The FIM improved significantly in both groups (RAT: Δ = 6.0 [2.0–16.5], *p* = 0.002; CR: Δ = 9.0 [3.5–12.5], *p* < 0.001), with no significant between-group difference in change (*p* = 0.940).

**Figure 3:**
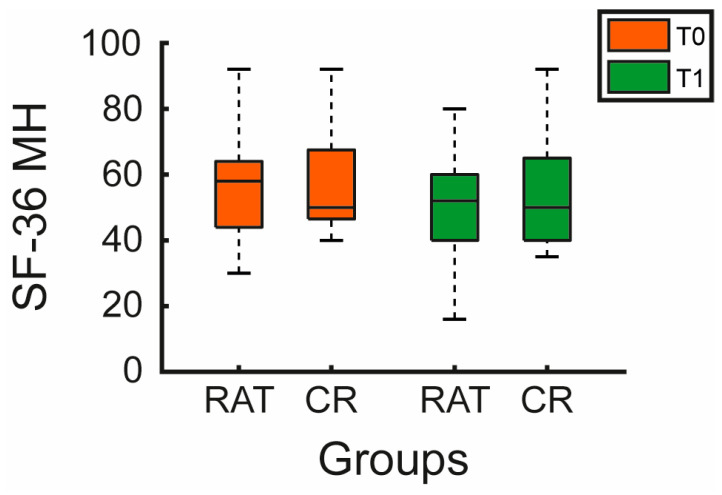
Mental Health outcomes. Boxplots display SF-36 Mental Health (SF-36 MH) scores for the experimental (RAT) and control (CR) groups at baseline (T0, orange) and post-intervention (T1, green). Boxes represent the interquartile range (Q1–Q3), the horizontal line marks the median, and whiskers extend to the most extreme observed values within 1.5 × interquartile range.

Across the SF-36 subscales, most changes were small and similar between groups. Physical Functioning (SF-36 PF) improved modestly in both groups (RAT: *p* = 0.005; CR: *p* = 0.016), with a slightly greater median change in the experimental group (*p* = 0.033 for Δ). General Health (SF-36 GH) decreased significantly in both groups (RAT: *p* = 0.017; CR: *p* < 0.001). Bodily Pain (SF-36 BP) improved significantly only in the control group (*p* = 0.006), while changes in the experimental group were not significant (*p* = 0.351). Mental Health (SF-36 MH) also showed a small but significant improvement in the control group (*p* = 0.037), but not in the experimental group (*p* = 0.069), as shown in Table 2 and Figure 3.

No significant between-group differences were found for any SF-36 domain (Δ comparisons). The summary of SF-36 indices confirmed these trends. The PCS decreased significantly within both groups (RAT: Δ = −5.1 [−8.4–1.9], *p* = 0.039; CR: Δ = −0.7 [−2.7–1.5], *p* = 0.408), though no significant between-group differences were detected (*p* = 0.283).

The MCS also showed a mild within-group decrease in the experimental group (Δ = −1.9 [−9.7–4.2], *p* = 0.234) and a similar trend in the controls (Δ = −1.5 [−4.5–1.4], *p* = 0.088). Although the change over time Δ did not differ significantly between groups (*p* = 0.692), the two groups differed significantly at T1 (*p* = 0.030), with the control group showing higher MCS values after treatment.

**Figure 4:**
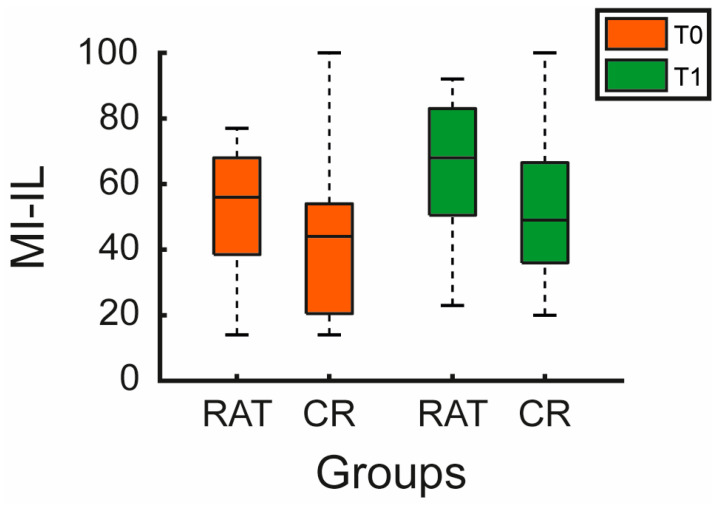
Motricity Index outcomes. Boxplots show Motricity Index impaired limb (MI-IL) scores for the experimental (RAT) and control (CR) groups at baseline (T0, orange) and post-intervention (T1, green). Boxes represent the interquartile range (Q1–Q3), the horizontal line marks the median, and whiskers extend to the most extreme observed values within 1.5 × interquartile range.

The MI for the impaired limb (MI-IL) improved significantly in both groups (RAT: Δ = 12.0 [9.0–16.0], *p* < 0.001; CR: Δ = 10.5 [0.0–19.0], *p* < 0.001, see Table 2 and Figure 4), with no significant between-group difference (*p* = 0.363). The MI for the unimpaired limb (MI-UL) remained at ceiling values (median 100 [100]) with no change (*p* = 1.000). See Table 2.

Both interventions led to significant reductions in pain and improvements in upper-limb motor performance. RAT produced outcomes comparable to those obtained with conventional treatment, indicating that it may promote functional recovery. Quality-of-life indices (PCS, MCS, and SF-36 domains) remained stable overall or showed small, similar variations across groups. Spasticity decreased slightly in the impaired limb, and no adverse effects were observed.

## 4. Discussion

This study evaluated whether RAT using a gravity-supporting exoskeleton (Armeo^®^ Spring) enhances upper-limb outcomes in chronic stroke with PSP versus dose-matched conventional therapy. A secondary aim was to examine functional and quality-of-life effects. We observed comparable improvements in pain, motor function, and functional independence across groups, while spasticity decreased only in the RAT group. These findings are consistent with the studies that found benefits to robotic-assisted rehabilitation.

### 4.1. Primary Outcomes

Regarding the primary outcomes, two complementary mechanisms may account for the observed reduction in spasticity with the RAT. First, high-repetition, task-specific training performed within safe kinematic ranges standardizes the dose and movement quality while limiting noxious end-range loading. Second, adjustable anti-gravity unloading optimizes scapula-humeral alignment, reducing abnormal synergies, and stretch-reflex recruitment. Consistent with this interpretation, Serrezuela et al. [26] reported that systematic anti-gravity training with an exoskeleton reduced spasticity in post-stroke patients and emphasized spasticity control as being central to mitigating shoulder pain. These factors likely contributed to the pain relief observed in our RAT group; however, similar pain improvements were also observed in the control group, and no statistically significant between-group differences were observed, suggesting comparable effects of RAT and CR for managing post-stroke pain in chronic patients.

Although our evaluation relied on clinical scales, rather than instrumented kinematics or electromyographic data, prior work indicates that reducing abnormal shoulder–elbow coupling can facilitate more effective movement after stroke [27]. Within this framework, the Armeo^®^ Spring’s graded weight support plausibly enables a practice that minimizes compensations; such a mechanism could help to explain the clinical gains in tone and pain we observed, even if changes in movement patterns were not directly measured in this study.

The benefits of gravity-support devices likely reflect their core training ingredients: intensive, repetitive, assisted-as-needed, and task-oriented practice [28,29,30]. Mechanically, the Armeo^®^ Spring provides adjustable arm-weight support through an elastic-band system [31]. Evidence indicates that weight-support training benefits subacute stroke patients with moderate-to-severe arm impairment, especially for vertical control (e.g., shoulder flexion) [32]. Finally, it is worth considering that robotic therapy provides 2D visual feedback, like a serious game. This type of feedback provides a training that is top-down, with an external focus of attention [33]. It is known that shifting the focus of attention can be an effective therapeutic strategy for increasing the range of motion and performance in patients with pain during rehabilitation [34,35].

Taken together, these results suggest that RAT with gravity support may represent a potential option to reduce tone and pain, consistent with previous results on chronic stroke [36]. Future studies should integrate objective kinematic and electrophysiological assessments to evaluate whether these mechanisms underlie the clinical benefits. Despite its positive effects, RAT should be considered a complementary, adjunctive approach to CR, rather than a substitute in current clinical practice. Robotic devices are costly and require specific staff training and regular maintenance. However, they may offer relevant practical advantages in selected settings, including the possibility of delivering high-intensity, repetitive, and standardized training, while also providing objective measures to monitor patients’ progress over time [37,38]. Nevertheless, the present study did not include a cost-effectiveness analysis; therefore, no conclusions can be drawn regarding the economic sustainability or comparative cost–benefit of RAT versus CR.

### 4.2. Secondary Outcomes

Regarding the secondary outcomes, both groups improved in MI, FIM, and health-related quality of life (SF-36). These findings are consistent with prior work in Parkinson’s disease, showing that Armeo^®^ Spring training can yield mobility gains relative to conventional physiotherapy [28]. It is noteworthy that RAT delivers intensive, task-oriented, assisted-as-needed practice that may engage activity-dependent plasticity and motor learning. Evidence from post-stroke cohorts indicates that such protocols can improve upper-limb dexterity and may modulate interhemispheric inhibitory dynamics [29,39]. Although our study used clinical scales rather than neurophysiological endpoints, these mechanisms may help to interpret the clinical changes observed; nevertheless, in the absence of neurophysiological measures, this remains speculative.

Notably, the magnitude of improvement in upper-limb mobility and functional independence in the RAT group was comparable to that in the conventional therapy group, as reported by other authors [40]; accordingly, no statistically significant between-group differences were observed for these outcomes between gravity-support exoskeleton training and conventional physiotherapy. While motor and functional outcomes improved, some SF-36 domains (particularly GH and PCS) evolved differently from objective clinical measures. This required careful interpretation, as the SF-36 captures perceived health status rather than functional capacity per se. In chronic neurological rehabilitation, increased use of the paretic limb and re-engagement in previously avoided activities may heighten awareness of residual motor limitations, fatigability, and movement inefficiency. The transition from learned non-use to active engagement can therefore coincide with a temporary decline in global health perception, despite measurable functional gains.

Moreover, improvements in mobility and independence may recalibrate patients’ expectations regarding participation and autonomy. When these expectations are only partially fulfilled, perceived physical health (GH and PCS) may transiently decrease. Thus, the SF-36 findings likely reflect an adaptation phase, rather than reduced therapeutic effectiveness. In this context, RAT may accelerate paretic limb re-use, amplifying this perception effect. Longitudinal follow-up studies are needed, as the perceived health status may improve once motor strategies consolidate and participation stabilizes.

From a cognitive–behavioral perspective, these patterns are compatible with graded exposure to movement, therapist feedback, and self-efficacy building [41]. These shared elements between groups reduce fear-avoidance and improve perceived function. Clinically, embedding brief pain education and graded exposure modules within RAT could enhance SF-36 BP without compromising RAT’s advantage in spasticity and movement quality [42]. Because SF-36 is a generic HRQoL instrument, domain-level changes may underrepresent limb-specific benefits; pairing it with upper-limb-specific HRQoL measures and cognitive–affective scales would better capture mechanisms and clinical relevance.

### 4.3. Limitations

This study has several limitations that should be acknowledged. First, its retrospective, single-center design based on routine clinical records limits causal inference and increases the risk of unmeasured confounding. Group allocation was not randomized and reflected standard care pathways; therefore, selection bias cannot be excluded. Second, outcome assessments were collected as part of usual care and extracted from medical charts, which may introduce information bias related to incomplete documentation and variability in assessment procedures. Third, the small sample size may limit the generalizability of the findings and the ability to detect subtle between-group differences. The study relied on clinical scales without objective kinematic or neurophysiological measures and lacked follow-up, preventing conclusions regarding underlying mechanisms and the durability of the observed effects.

Finally, the retrospective design did not allow for control over assessor blinding. As pain and spasticity are partially subjective outcomes, the potential influence of evaluator awareness of treatment allocation cannot be excluded.

## 5. Conclusions

In patients with chronic stroke and post-stroke pain, robotic-assisted upper limb training with a gravity-support exoskeleton (Armeo^®^ Spring) was associated with reduced spasticity. For pain, upper-limb mobility, and functional independence, no statistically significant between-group differences were observed compared with conventional physiotherapy. Overall, our findings suggest that gravity-supported RAT may be associated with improvements in tone and movement quality in chronic stroke. However, given the retrospective design and the sample size, these observations should be interpreted as exploratory and confirmed in larger prospective randomized trials. Future studies should pair clinical scales with instrumental kinematics and neurophysiology and include longer follow-ups to clarify the mechanisms and durability.

## Figures and Tables

**Table 1:** Baseline demographic and clinical characteristics of the study population. Data are presented as mean ± standard deviation (SD) for continuous variables and as counts (percentage) for categorical variables. Group comparisons were performed using independent-sample *t*-tests or Mann–Whitney U tests for continuous variables, and chi-squared test (χ^2^) or Fisher’s exact tests (Fisher) for categorical variables. No significant differences were observed between groups at baseline (*p* > 0.05 for all).

	All	RAT	CR	*p*-Value	Test Type
Age	58 ± 12.9	58.4 ± 14.6	57.6 ± 11.4	0.862	*t*-test (equal variances assumed)
MoCA	23.1 ± 3.2	23.1 ± 3.3	23 ± 3.2	0.914	*t*-test (equal variances assumed)
Gender				1	Fisher
Male (%)	24 (75)	12 (75)	12 (75)		
Female (%)	8 (25)	4 (25)	4 (25)		
Etiology				0.988	χ^2^
Hemorrhagic (%)	10 (31.25)	6 (37.5)	4 (25)		
Ischemic (%)	22 (68.75)	10 (62.5)	12 (75)		
Education	9.6 ± 2.5	9.7 ± 2.8	9.6 ± 2.4	0.929	Mann–Whitney (Wilcoxon rank-sum)
Year Since Disease	1.5 ± 0.6	1.6 ± 0.6	1.5 ± 0.6	0.766	Mann–Whitney (Wilcoxon rank-sum)
Affected side				0.5582	χ^2^
Right (%)	19 (59.38)	8 (50)	11 (68.75)		
Left (%)	13 (40.63)	8 (50)	5 (31.25)		

Legend: MoCA = Montreal Cognitive Assessment.

**Table 2:** Clinical outcome measures at baseline (T0) and after treatment (T1) in the experimental group (RAT), and the control group (CR). Data are presented as the median [interquartile range (Q1–Q3)]. Δ represents the change score (T1–T0). Within-group comparisons (T0 vs. T1) were performed using the Wilcoxon signed-rank test. Between-group comparisons (RAT vs. CR) at T0 and T1 and for Δ were conducted using the Mann–Whitney U test. *p*-values < 0.05 are shown in bold.

Field	RAT				CR				Between Groups (RAT vs. CR)		
	T0 Med [Q1–Q3]	T1 Med [Q1–Q3]	Δ Med [Q1–Q3]	P Intra	T0 Med [Q1–Q3]	T1 Med [Q1–Q3]	Δ Med [Q1–Q3]	P Intra	P (T0)	P (T1)	P (Δ)
NPRS	4.5 [3.0–6.0]	2.5 [0.0–4.0]	−2.0 [−3.5–−1.5]	<0.001	4.0 [3.0–6.0]	2.0 [1.0–4.0]	−2.0 [−3.0–−1.0]	0.004	1.000	0.603	0.437
FIM	91.5 [83.0–100.5]	101.0 [93.0–116.0]	6.0 [2.0–16.5]	**0.002**	100.0 [81.0–109.5]	110.0 [89.5–115.0]	9.0 [3.5–12.5]	**<0.001**	0.485	0.970	0.940
SF-36 PF	37.5 [10.0–70.0]	2.5 [0.0–22.5]	−10.0 [−35.0–−2.5]	**0.005**	5.0 [0.0–46.5]	0.0 [0.0–27.5]	0.0 [−5.0–0.0]	**0.016**	0.100	0.803	**0.033**
SF-36 RLPH	0.0 [0.0–25.0]	0.0 [0.0–5.0]	0.0 [−12.5–0.0]	0.219	0.0 [0.0–10.0]	0.0 [0.0–0.0]	0.0 [0.0–0.0]	0.250	0.948	0.774	0.601
SF-36 BP	51.5 [40.5–80.0]	46.0 [25.0–69.0]	−10.5 [−33.5–19.5]	0.351	79.5 [57.5–95.0]	73.0 [35.5–92.0]	−4.5 [−14.5–0.0]	**0.006**	0.056	0.107	0.791
SF-36 GH	57.5 [32.5–73.5]	33.5 [25.0–45.0]	−20.0 [−38.5–−5.0]	**0.017**	37.5 [32.5–52.5]	33.5 [25.0–40.0]	−8.0 [−12.5–−2.5]	**<0.001**	0.156	0.746	0.072
SF-36 VT	45.0 [30.0–60.0]	37.5 [30.0–55.0]	−7.5 [−25.0–10.0]	0.211	52.5 [40.0–62.5]	55.0 [37.5–60.0]	−5.0 [−5.0–5.0]	0.848	0.298	0.098	0.358
SF-36 SF	50.0 [25.0–50.0]	31.0 [12.5–56.0]	0.0 [−25.0–2.5]	0.564	55.0 [27.5–75.0]	62.0 [25.0–75.0]	−1.5 [−6.5–7.0]	1.000	0.198	0.108	0.334
SF-36 RLEP	33.0 [0.0–100.0]	68.0 [0.0–100.0]	0.0 [−15.0–17.0]	0.594	50.0 [17.5–87.5]	60.0 [15.0–100.0]	0.0 [−1.0–0.5]	0.672	0.862	0.922	1.000
SF-36 MH	58.0 [44.0–64.0]	52.0 [40.0–60.0]	−7.0 [−12.0–4.0]	0.069	50.0 [46.5–67.5]	50.0 [40.0–65.0]	−3.5 [−5.0–0.0]	**0.037**	0.940	0.835	0.316
PHC	32.0 [28.4–44.2]	28.9 [26.9–32.5]	−5.1 [−8.4–1.9]	**0.039**	29.2 [24.0–33.6]	27.7 [23.8–33.0]	−0.7 [−2.7–1.5]	0.408	0.220	0.509	0.283
MHC	39.0 [36.1–50.9]	39.6 [35.7–45.1]	−1.9 [−9.7–4.2]	0.234	46.5 [44.8–52.7]	48.2 [40.6–50.8]	−1.5 [−4.5–1.4]	0.088	0.086	**0.030**	0.692
MI-IL	56.0 [38.5–68.0]	68.0 [50.5–83.0]	12.0 [9.0–16.0]	**<0.001**	44.0 [20.5–54.0]	49.0 [36.0–66.5]	10.5 [0.0–19.0]	**<0.001**	0.104	0.070	0.363
MI-UL	100.0 [100.0–100.0]	100.0 [100.0–100.0]	0.0 [0.0–0.0]	1.000	100.0 [100.0–100.0]	100.0 [100.0–100.0]	0.0 [0.0–0.0]	1.000	1.000	0.349	0.349
MAS-IL	1.0 [0.0–3.0]	1.0 [0.0–2.5]	0.0 [−1.0–0.0]	**0.031**	3.0 [1.0–3.0]	1.5 [0.0–3.0]	0.0 [−1.5–0.0]	0.102	0.559	0.545	0.818
MAS-UL	0.0 [0.0–0.0]	0.0 [0.0–0.0]	0.0 [0.0–0.0]	1.000	0.0 [0.0–0.0]	0.0 [0.0–0.0]	0.0 [0.0–0.0]	1.000	0.349	0.349	1.000

Legend: NPRS = Numeric Pain Rating Scale; FIM = Functional Independence Measure; SF-36 PF = Short Form Health Survey 36 Physical Functioning; SF-36 RLPH = Short Form Health Survey 36 Role Limitation due to Physical Health; SF-36 BP = Short Form Health Survey 36 Bodily Pain; SF-36 GH = Short Form Health Survey 36 General Health perception; SF-36 VT = Short Form Health Survey 36 Vitality; SF-36 SF = Short Form Health Survey 36 Social Functioning; SF-36 RLEP = Short Form Health Survey 36 Role Limitation due to Emotional Problems; SF-36 MH = Short Form Health Survey 36 Mental Health; PHC = Physical Health Component; MHC = Mental Health Component; MI-IL = Motricity Index impaired limb; MI-UL = Motricity Index unimpaired limb; MAS-IL = Modified Ashworth Scale impaired limb; MAS-UL = Modified Ashworth Scale unimpaired limb.

## Data Availability

The data presented in this study are available upon request from the corresponding author, due to the privacy of research participants.

## Abbreviations

The following abbreviations are used in this manuscript:

BP	Bodily Pain
CNS	Central Nervous System
CR	Conventional Rehabilitation
FIM	Functional Independence Measure
GH	General Health
HRQoL	Health-Related Quality of Life
IL	Impaired Limb
MAS	Modified Ashworth Scale
MCS	Mental Component Summary
MH	Mental Health
MI	Motricity Index
MoCA	Montreal Cognitive Assessment
NPRS	Numeric Pain Rating Scale
PCS	Physical Component Summary
PF	Physical Functioning
PSP	Post-Stroke Pain
Q1	First quartile (25th percentile)
Q3	Third quartile (75th percentile)
RAT	Robotic-Assisted Therapy
RLEP	Role Limitation due to Emotional Problems
RLPH	Role Limitation due to Physical Health
SD	Standard Deviation
SF	Social Functioning
SF-36	Short Form-36 Health Survey
T0	Before Treatment
T1	After Treatment
UL	Unimpaired Limb
VR	Virtual Reality
VT	Vitality

## References

[1] Feigin V.L., Stark B.A., Johnson C.O., Roth G.A., Bisignano C., Abady G.G., Abbasifard M., Abbasi-Kangevari M., Abd-Allah F., Abedi V. (2021). Global, Regional, and National Burden of Stroke and Its Risk Factors, 1990–2019: A Systematic Analysis for the Global Burden of Disease Study 2019. Lancet Neurol..

[2] Wafa H.A., Wolfe C.D.A., Emmett E., Roth G.A., Johnson C.O., Wang Y. (2020). Burden of Stroke in Europe. Stroke.

[3] Murphy S.J.X., Werring D.J. (2020). Stroke: Causes and Clinical Features. Medicine.

[4] O’Donnell M.J., Diener H.-C., Sacco R.L., Panju A.A., Vinisko R., Yusuf S., On Behalf of PRoFESS Investigators (2013). Chronic Pain Syndromes After Ischemic Stroke. Stroke.

[5] Appelros P. (2006). Prevalence and Predictors of Pain and Fatigue after Stroke: A Population-Based Study. Int. J. Rehabil. Res..

[6] Lundström E., Smits A., Terént A., Borg J. (2009). Risk Factors for Stroke-Related Pain 1 Year after First-Ever Stroke. Eur. J. Neurol..

[7] Tang W.K., Liang H., Mok V., Ungvari G.S., Wong K.-S. (2013). Is Pain Associated with Suicidality in Stroke?. Arch. Phys. Med. Rehabil..

[8] Harrison R.A., Field T.S. (2015). Post Stroke Pain: Identification, Assessment, and Therapy. Cerebrovasc. Dis..

[9] Paolucci S., Iosa M., Toni D., Barbanti P., Bovi P., Cavallini A., Candeloro E., Mancini A., Mancuso M., Monaco S. (2016). Prevalence and Time Course of Post-Stroke Pain: A Multicenter Prospective Hospital-Based Study. Pain Med..

[10] Lan Nguyen Hoang C., Salle J.-Y., Mandigout S., Hamonet J., Macian-Montoro F., Daviet J.-C. (2012). Physical Factors Associated with Fatigue After Stroke: An Exploratory Study. Top. Stroke Rehabil..

[11] Torres-Parada M., Vivas J., Balboa-Barreiro V., Marey-López J. (2020). Post-Stroke Shoulder Pain Subtypes Classifying Criteria: Towards a More Specific Assessment and Improved Physical Therapeutic Care. Braz. J. Phys. Ther..

[12] Roosink M., Renzenbrink G.J., Geurts A.C.H., Ijzerman M.J. (2012). Towards a Mechanism-Based View on Post-Stroke Shoulder Pain: Theoretical Considerations and Clinical Implications. NeuroRehabilitation.

[13] Wissel J., Schelosky L.D., Scott J., Christe W., Faiss J.H., Mueller J. (2010). Early Development of Spasticity Following Stroke: A Prospective, Observational Trial. J. Neurol..

[14] de Sire A., Moggio L., Demeco A., Fortunato F., Spanò R., Aiello V., Marotta N., Ammendolia A. (2022). Efficacy of Rehabilitative Techniques in Reducing Hemiplegic Shoulder Pain in Stroke: Systematic Review and Meta-Analysis. Ann. Phys. Rehabil. Med..

[15] Marque P., Gasq D., Castel-Lacanal E., De Boissezon X., Loubinoux I. (2014). Post-Stroke Hemiplegia Rehabilitation: Evolution of the Concepts. Ann. Phys. Rehabil. Med..

[16] Molteni F., Gasperini G., Cannaviello G., Guanziroli E. (2018). Exoskeleton and End-Effector Robots for Upper and Lower Limbs Rehabilitation: Narrative Review. PM&R.

[17] van Vliet P.M., Wulf G. (2006). Extrinsic Feedback for Motor Learning after Stroke: What Is the Evidence?. Disabil. Rehabil..

[18] Mancuso M., Iosa M., Morone G., De Bartolo D., Ciancarelli I. (2025). How Do the Timing of Early Rehabilitation Together with Cognitive and Functional Variables Influence Stroke Recovery? Results from the CogniReMo Italian Multicentric Study. Healthcare.

[19] Chan S.C.C., Chan C.C.H., Kwan A.S.K., Ting K., Chui T. (2012). Orienting Attention Modulates Pain Perception: An ERP Study. PLoS ONE.

[20] Gnasso R., Palermi S., Picone A., Tarantino D., Fusco G., Messina M.M., Sirico F. (2023). Robotic-Assisted Rehabilitation for Post-Stroke Shoulder Pain: A Systematic Review. Sensors.

[21] Rodriguez C.S. (2001). Pain Measurement in the Elderly: A Review. Pain Manag. Nurs..

[22] Harb A., Margetis K., Kishner S. (2025). Modified Ashworth Scale. StatPearls.

[23] Longo D., Doronzio S., Piazzini M., Politi A.M., Ciapetti T., Gerli F., Barnabé M., Ciullini F., Castagnoli C., Pellegrini I. (2025). Development of the Italian version of the motricity index and evaluation of its reliability in adults with stroke. J. Rehabil. Med..

[24] Keith R.A., Granger C.V., Hamilton B.B., Sherwin F.S. (1987). The Functional Independence Measure: A New Tool for Rehabilitation. Adv. Clin. Rehabil..

[25] Brazier J.E., Harper R., Jones N.M., O’Cathain A., Thomas K.J., Usherwood T., Westlake L. (1992). Validating the SF-36 Health Survey Questionnaire: New Outcome Measure for Primary Care. BMJ.

[26] Serrezuela R.R., Quezada M.T., Zayas M.H., Pedrón A.M., Hermosilla D.M., Zamora R.S. (2020). Robotic Therapy for the Hemiplegic Shoulder Pain: A Pilot Study. J. Neuroeng. Rehabil..

[27] Beer R.F., Ellis M.D., Holubar B.G., Dewald J.P.A. (2007). Impact of gravity loading on post-stroke reaching and its relationship to weakness. Muscle Nerve.

[28] Raciti L., Pignolo L., Perini V., Pullia M., Porcari B., Latella D., Isgrò M., Naro A., Calabrò R.S. (2022). Improving Upper Extremity Bradykinesia in Parkinson’s Disease: A Randomized Clinical Trial on the Use of Gravity-Supporting Exoskeletons. J. Clin. Med..

[29] Calabrò R.S., Naro A., Russo M., Leo A., De Luca R., Balletta T., Buda A., La Rosa G., Bramanti A., Bramanti P. (2017). The Role of Virtual Reality in Improving Motor Performance as Revealed by EEG: A Randomized Clinical Trial. J. Neuroeng. Rehabil..

[30] Morone G., Tramontano M., Paolucci S., Cerasa A., Ciancarelli I., Martino Cinnera A., Iosa M., Calabrò R.S. (2025). Tailoring Robot-Assisted Arm Training to Individuals with Stroke: Bridging Neuroscience Principles and Clinical Practice. Front. Neurol..

[31] Perry B.E., Evans E.K., Stokic D.S. (2017). Weight Compensation Characteristics of Armeo^®^Spring Exoskeleton: Implications for Clinical Practice and Research. J. Neuroeng. Rehabil..

[32] Chan I.H.L., Fong K.N.K., Chan D.Y.L., Wang A.Q.L., Cheng E.K.N., Chau P.H.Y., Chow K.K.Y., Cheung H.K.Y. (2016). Effects of Arm Weight Support Training to Promote Recovery of Upper Limb Function for Subacute Patients after Stroke with Different Levels of Arm Impairments. BioMed Res. Int..

[33] Morone G., Ghanbari Ghooshchy S., Palomba A., Baricich A., Santamato A., Ciritella C., Ciancarelli I., Molteni F., Gimigliano F., Iolascon G. (2021). Differentiation among Bio- and Augmented- Feedback in Technologically Assisted Rehabilitation. Expert Rev. Med. Devices.

[34] Wulf G. (2007). Attention and Motor Skill Learning.

[35] Kim G.J., Hinojosa J., Rao A.K., Batavia M., O’Dell M.W. (2017). Randomized Trial on the Effects of Attentional Focus on Motor Training of the Upper Extremity Using Robotics with Individuals After Chronic Stroke. Arch. Phys. Med. Rehabil..

[36] Morone G., Capone F., Iosa M., Cruciani A., Paolucci M., Martino Cinnera A., Musumeci G., Brunelli N., Costa C., Paolucci S. (2022). May Dual Transcranial Direct Current Stimulation Enhance the Efficacy of Robot-Assisted Therapy for Promoting Upper Limb Recovery in Chronic Stroke?. Neurorehabilit. Neural Repair.

[37] Banyai A.D., Brișan C. (2024). Robotics in Physical Rehabilitation: Systematic Review. Healthcare.

[38] Calabrò R.S., De Cola M.C., Leo A., Reitano S., Balletta T., Trombetta G., Naro A., Russo M., Bertè F., De Luca R. (2015). Robotic Neurorehabilitation in Patients with Chronic Stroke: Psychological Well-Being beyond Motor Improvement. Int. J. Rehabil. Res..

[39] Calabrò R.S., Accorinti M., Porcari B., Carioti L., Ciatto L., Billeri L., Andronaco V.A., Galletti F., Filoni S., Naro A. (2019). Does Hand Robotic Rehabilitation Improve Motor Function by Rebalancing Interhemispheric Connectivity after Chronic Stroke? Encouraging Data from a Randomised-Clinical-Trial. Clin. Neurophysiol..

[40] Taveggia G., Borboni A., Salvi L., Mulé C., Fogliaresi S., Villafañe J.H., Casale R. (2016). Efficacy of Robot-Assisted Rehabilitation for the Functional Recovery of the Upper Limb in Post-Stroke Patients: A Randomized Controlled Study. Eur. J. Phys. Rehabil. Med..

[41] Pinto Barbosa S., Marques L., Sugawara A., Toledo F., Imamura M., Battistella L., Simis M., Fregni F. (2022). Predictors of the Health-Related Quality of Life (HRQOL) in SF-36 in Knee Osteoarthritis Patients: A Multimodal Model with Moderators and Mediators. Cureus.

[42] Schemer L., Schroeder A., Ørnbøl E., Glombiewski J.A. (2019). Exposure and Cognitive-Behavioural Therapy for Chronic Back Pain: An RCT on Treatment Processes. Eur. J. Pain.

